# "Styptic Colloid." A New Styptic and Adhesive Fluid

**Published:** 1867-07

**Authors:** Benjamin W. Richardson

**Affiliations:** Senior Physician to the Royal Infirmary for Diseases of the Chest.


### ARTICLE VI.

### "*Styptic Colloid.*" *A New Styptic and Adhesive Fluid.*

By Benjamin W. Richardson, M. A., M. D., F. R. C. P., Senior Physician to the Royal Infirmary for Diseases of the Chest.

"The use of collodion, or gun-cotton dissolved in ether, had for some years been recognized as of great service in covering wounds, and as I found that collodion was very easily diffusible in the form of spray, it seemed to me that it might be applied in that form with advantage, and might even be made to coat a bleeding open surface. But on experiment it came out that in whatever way applied, collodion fulfils but a small part of the required duty as a styptic and a wound-healer. In the first place, it mixes indifferently with blood ; in the second place, it forms but an imperfect adhesion ; and lastly, it allows the transudation of air through its structure when laid upon a moist surface, and particularly upon the body, which is at all times throwing off watery vapor. At the same time the principle of the collodion process was excellent: to lay down from a fluid a deposit of solid matter by the evaporation of a volatile solvent was sound and beautiful action.

"The next point in advance, therefore, was to combine, if possible, with the ether and gun-cotton some other substance which being also soluble in ether and capable of deposit by the evaporation of the ether, would combine chemically with the blood, with the albuminous exudative matter of a wound, or with purulent matter.

"The idea only was wanted to secure the object in view. There was one substance which answered all these indications—I mean tannin. A mixture, therefore, of xyloidine, a substance resembling gun-cotton, and of tannin was formed into solution with ether, and from that came what I designated '*xylo-styptic ether.*'

"Brought into practice, the advantages of this solution as a means for stopping hæmorrhage at once became obvious. Indeed, as a means for the arrest of hæmorrhage, less than the application of a ligature to an open vessel, the spray leaves little to be desired. The extreme cold produced by the evaporation of the ether acts directly on the water of the blood, the tannin solidifies the blood by combining with it, and the cotton acts as a plug: thus every indication for arrest of hæmorrhage is secured.

"But in observing the action of the styptic spray, I soon became impressed with another fact, viz. : that after the application to decomposing and fetid wounds and sores, the fetor entirely disappeared, the wounds commenced to heal with great rapidity, and a kind of natural covering appeared to form out of the secretion by its combination with the dressing above it.

"This observation has led me to simplify the application still further until it has come into this convenient form, a mere solution capable of being kept on the table as gum is kept, and of being applied with a soft brush in the same simple way.

"The process of manufacture of the fluid is tedious, but sufficiently easy. The object to be aimed at is to saturate ether entirely with tannin and a colloidal substance, xyloidine or gun-cotton. In the first step of the process, the tannin, rendered as pure as it can be, is treated with absolute alcohol, and is made to digest in the alcohol for several days. Then the ether, also absolute, is added until the whole of the thick alcoholic mixture is rendered quite fluid. Next the colloidal substance is put in until it ceases readily to dissolve. For the sake of its very agreeable odor, a little tincture of benzoin is finally admixed.

"The solution is now ready for use. It can be applied directly with a brush, or mixed with equal quantities of ether, it can be applied in the form of a spray. In order to give to the fluid a short name by which it may be known, I have called it '*styptic colloid.*'

"*Properties.* When the solution is brought into contact with an open surface of the body, the resultant phenomena are these: the heat of the body gradually volatilizes the ether and the alcohol, and the tannin and cotton, as the ether leaves them, are thus left stranded on the surface in intimate combination. In proportion as the ether passes off, the blood or the secretion of the surface permeates the tannin and cotton; but tannin acts directly upon albumen, coagulating it, and transforming it into a kind of membrane, almost like leather. The cotton meanwhile unites the whole, gives substance to the mass, and adhesive quality. When all is solidified, the dressing becomes, in fact, a concrete, having a true organic hold or basis on the tissue ; and as the tannin, if the solution be freely applied, is in excess, any new exudative matter or blood is for several hours taken up by it, and the annealing is made the more complete.

"Thus, by this dressing, the air is excluded from every possible point in every possible direction, not by a mere septum, but by the combination of the animal fluids with the remedy ; and because the air is excluded and fluid is absorbed there is no decomposition—*i.e.* no oxidation ; and because there is no oxidation there is no irritation.

"The styptic and adhesive qualities of this fluid are easily demonstrated by observing its direct action on blood on serum, on pus, on albumen. You will see that it solidifies all these by mere contact with them.

"To these properties I must also add that of complete deodorization. Here is putrid blood, here putrid ovarian serum, here putrid purulent substance. They are unapproachable when laid on an open surface, but we bring them into contact with the solution, and they are deodorized. Further, the decomposed substance is fixed by the tannin and rendered inert.

"*Modes of Application.* Having thus laid down the principles at work in this method of treatment, let me next pass to the modes of application. I have here an artificial limb; a portion of it is covered with thin skin to represent the ordinary skin. At one part, for the space of six inches by four, there is placed under the skin a spongy substance charged with blood and albuminous fluid. I will now make an incision through the skin, and expose the bleeding surface. Suppose this to be an open wound, the two flaps of an amputation. I close it with silk ligatures in five places. This done, I take a little cotton-wool, tease it out finely in a wineglass, and saturate it with the styptic solution. Next, with a soft camel-hair brush, I apply the solution freely over the closed wound, letting it lie between the edges. If blood exude, it simply combines with the solution, making a mass much like red wax. I lay on the solution also for a little distance beyond the wound, and wait a few moments to allow for the evaporation of ether. Next I take from the wineglass the saturated cotton-wool with forceps, and lay a seam of it half an inch wide and the eighth of an inch in thickness over the line of incision. Finally, I coat the whole over with another layer of the solution, and wait until the layer is nearly dry, cover with a little dry cotton, and, if pressure be necessary, carry over the whole a bandage.

"If time is a matter of importance, the evaporation of the fluid can be hastened by gently blowing with the warm breath over the solution as each layer of it is applied with the brush.

"Presuming that a cavity has to be treated, the fluid is often more neatly and handily used as spray. Thus, in treating the roof of the mouth for carious bone, or in plugging a bleeding alveolar cavity after extraction of a tooth, the spray is excellent. We begin in such a case by applying the spray direct to the bleeding surface, and when a layer of deposit is formed we use that as a foundation for a thin layer of cotton-wool ready saturated in the solution. Then we reapply the spray, and again cotton, until the whole operation is complete.

"For bleeding or fetid discharge from the uterus or vagina the spray is not advisable, because of the introduction of air. Here injection by the syringe is the best process, followed, if need be, by a plug of cotton-wool saturated with the solution.

"In cases of compound fracture, after the parts have been brought into apposition as far as is possible and fixed in the necessary position the fluid should be poured slowly into the open cavity so as to fill it. Then the parts externally should be covered with a layer of cotton-wool saturated with the solution.

"On open cancer, and on suppurating or decomposing surfaces, the solution may be freely applied with the brush, and afterward the parts may be covered with cotton-wool saturated with the fluid.

"In no case need there be any fear that irritation will follow the application of the solution. On the contrary, the action of it is so purely negative that it might be considered a sedative. It is not such in the technical sense of the term, but it so effectually covers the wounded and susceptible surfaces as to maintain what is virtually a sedative influence.

"After a fresh wound has been once dressed with this solution, it requires but little further treatment. In the case of small wounds they may be safely left with one dressing. In process of cure the dressing will slowly be thrown off in the form of a thick scale, and ligatures will also spontaneously come away. Even when the wound is very large, as after amputation, it is not desirable to try to open the wound unless there be systemic symptoms. In such case, in order to remove the dressing without pain, the bandage, if it be adherent, must be sponged at the adherent parts with a mixture of alcohol and ether, or with alcohol and water; this will set everything at liberty with ease and cleanliness. Water alone must on no account be used, neither hot nor cold.

"THE FLUID IN PRACTICE.—From these directions I may now move to the recital of a few typical cases in which this solution has been employed.

"*Hæmorrhage after Tooth Extraction.* At the request of my friend, Mr. Thomas A. Rogers, of Hanover Square, I was summoned on May 9th, 1866, at 10 A. M., to see a young gentleman who was suffering from a dangerous hæmorrhage from an alveolar cavity from which a large molar tooth had been extracted. Mr. Rogers had extracted the tooth at 5 o'clock on the previous evening, and from then until my arrival the bleeding had never ceased. I learned from the parents that all their children suffered from the hæmorrhagic tendency in an extreme degree. The young man was now in a state of very great exhaustion, and the flow of fluid blood was still continuous. Laying him upon the floor, with his head raised, I applied the styptic, in the form of spray, directly into the bleeding cavity, and with such force at first as to dislodge the little pool of blood there. Continuing the spray, I was soon gratified at seeing complete arrest of the hæmorrhage. I now placed in the cavity a small pledget of cotton-wool saturated with the fluid, pressed it firmly home, and covered it with spray; thus, layer upon layer, I not only filled the cavity with cotton-wool and firmly-set styptic, but builded up the space between the two adjoining teeth with the same substances until the gap in the middle looked as if filled with what the dentists call a bone block. The bleeding was entirely arrested from that moment, and recovery was perfect. There was no sign of irritation in the wound, and when, four days afterward, the plug came away, all the structures beneath were healed.

"*Severe Hemorrhage from Necrosis of the Upper Maxilla.* A gentleman suffering ftom syphilis, extremely aggravated by excess of mercury, was last year brought under the care of my friends Messrs. Musgrave and Milson, of St. John's-wood. I saw the patient with them in consultation many times, and were all in much anxiety in regard to the roof of the mouth. The mucus membrane of the roof was thickened, red, percolated, and offensive; the fetor was almost unbearable, and every evidence of necrosed bone was present. The soft covering of the palate at length gave way, and the soft parts ulcerated, and about midnight on October 16, the necrosed bone loosened, and was partly displaced. In this way, the two descending palatine arteries were severed, and the most sudden and profuse hæmorrhage followed. I was summoned, and soon after my arrival, Mr. Milson also came. Unfortunately, we had to send some distance for the styptic fluid, but meanwhile we tried, quite ineffectually, to plug with the perchloride of iron, and were compelled to trust to pressure with the fingers. When the styptic arrived, we played it freely in the form of spray over the bleeding points, and with instant effect in stopping the loss of blood. Then we plugged carefully with cotton, saturating each layer with the spray, and soon the mass set firmly, and no more blood was lost. By the time we had controlled the bleeding, we were gratified by the arrival of Mr. Paget, whom we had summoned ; and as he, from having already tried the styptic, was content to trust to it, we did no more. It is the fact that this patient lost five pints of blood before the fluid was used, but the hæmorrhage did not return, the fetor no longer was present : and a few days later, assisted by Mr. Milson, I removed the dead bone. The removal laid bare a wide ulcerated surface or cavity, which we continued to plug with the styptic or cotton-wool ; and so perfect has been the healing process that the cavity would now bear an artificial plate. —*Med. Times and Gazette.*

---

